# Patterns of Novel Alleles and Genotype/Phenotype Correlations Resulting from the Analysis of 108 Previously Undetected Mutations in Patients Affected by Neurofibromatosis Type I

**DOI:** 10.3390/ijms18102071

**Published:** 2017-09-29

**Authors:** Francesco Bonatti, Alessia Adorni, Annalisa Matichecchia, Paola Mozzoni, Vera Uliana, Francesco Pisani, Livia Garavelli, Claudio Graziano, Maria Gnoli, Diana Carli, Stefania Bigoni, Elena Boschi, Davide Martorana, Antonio Percesepe

**Affiliations:** 1 Medical Genetics, University Hospital of Parma, 43126 Parma, Italy; fra.bonatti@gmail.com (F.B.); nalessia.tin@gmail.com (A.A.); annalisa.matichecchia@gmail.com (A.M.); paola.mozzoni@unipr.it (P.M.); vuliana@ao.pr.it (V.U.); dmartorana@ao.pr.it (D.M.); 2 Children’s Neuropsycological Services, University Hospital of Parma, 43126 Parma, Italy; francesco.pisani@unipr.it; 3 Clinical Genetics, IRCCS S. Maria Nuova Hospital, Reggio 42123 Emilia, Italy; Livia.Garavelli@asmn.re.it; 4 Medical Genetics, S. Orsola-Malpighi University Hospital, 40138 Bologna, Italy; claudio.graziano@unibo.it; 5 Medical Genetics and Skeletal Rare Diseases, Istituto Ortopedico Rizzoli, 40126 Bologna, Italy; maria.gnoli@ior.it; 6 Medical Genetics, Città della Salute e della Scienza University Hospital, 10126 Torino, Italy; diana.carli@unito.it; 7 UOL Medical Genetics, University of Ferrara, 44121 Ferrara, Italy; stefania.bigoni@unife.it; 8 Plastic Surgery, University Hospital of Parma, 43126 Parma, Italy; eboschi@ao.pr.it

**Keywords:** neurofibromatosis type I, *NF1* gene, genotype/phenotype correlation

## Abstract

Neurofibromatosis type I, a genetic disorder due to mutations in the *NF1* gene, is characterized by a high mutation rate (about 50% of the cases are de novo) but, with the exception of whole gene deletions associated with a more severe phenotype, no specific hotspots and few solid genotype/phenotype correlations. After retrospectively re-evaluating all *NF1* gene variants found in the diagnostic activity, we studied 108 patients affected by neurofibromatosis type I who harbored mutations that had not been previously reported in the international databases, with the aim of analyzing their type and distribution along the gene and of correlating them with the phenotypic features of the affected patients. Out of the 108 previously unreported variants, 14 were inherited by one of the affected parents and 94 were de novo. Twenty-nine (26.9%) mutations were of uncertain significance, whereas 79 (73.2%) were predicted as pathogenic or probably pathogenic. No differential distribution in the exons or in the protein domains was observed and no statistically significant genotype/phenotype correlation was found, confirming previous evidences.

## 1. Introduction

The rapid evolution of the high-throughput sequencing technologies presents significant challenges in the acquisition, analysis and interpretation of large data sets. In particular, the detection of several novel alleles in multiple-gene panels or clinical exomes brings about difficulties in the understanding of the significance of the variants found and finally in the interpretation and clinical use of the genetic result in settings like pre-symptomatic or prenatal diagnosis [1,2]. Disease-specific databases, knowledge of the allele frequencies and the type of nucleotide change help to sort single variants into benign or pathogenic, but some of them remain uncertain about their clinical significance [3], especially when they are found in genes causing very rare diseases or those with a reduced penetrance or with a high mutation rate [4]. The approach to the interpretation of single variants used for genes such as *NF1* or *BRCA1* [1,5], based on a distribution into classes of growing evidence of pathogenicity (five, at present), has then been translated to the interpretation of the results of clinical exomes or large gene-panels.

In addition to providing a strategy for the clinical use of new gene variants, *NF1* (MIM*613113) is a model gene for the high rate of new alleles (about 50% of the total burden of mutations [6]) and for their fully penetrant mendelian behavior, which makes them detectable in specific phenotypes belonging to neurofibromatosis type I (NF1) (MIM#162200) (various combinations of multiple café-au-lait spots, axillary and inguinal freckling, multiple neurofibromas, and iris Lisch nodules) [7]. Understanding the features of the *NF1* novel variants can shed light on the patterns of genome variability, natural selection, and evolution.

Moreover, the clinical phenotypic expression characterized by marked intra- and inter-familial variability with multisystemic complications including neurological, cardiovascular, gastrointestinal, endocrine, neoplastic, and orthopedic features [8] has long puzzled physicians and researchers in the attempt to predict which genotypes would harbor the highest risk of complications. Very few, however, are the established genotype–phenotype correlations, whose best example is represented by the *NF1* extended deletions, which have been linked to a more severe phenotype than point mutations [9,10,11]. Finally, no evidence exists demonstrating that mutations cluster in specific gene regions or protein domains of which the NF1 protein, neurofibromin, is composed, including a cysteine–serine-rich domain (CSRD), a tubulin-binding domain (TBD), a central GTPase-activating protein-related (GRD), a SEC14 domain, a syndecan binding 1 (SH1) domain, a pleckstrin homology (PH) domain, a syndecan binding 2 (SH2) domain, and a carboxy-terminal domain (CTD) [12].

In the present study, we retrospectively re-evaluated all *NF1* gene variants found in 17 years of diagnostic activity and selected all the mutations not reported in the international databases or in the medical literature. Those latter were stratified according to the five pathogenetic classes, analyzed for their type and their distribution in the exons of the *NF1* gene and in the domains of the relative protein. Finally, after dividing the phenotypic features of the disease into cardinal signs and complications, a genotype/phenotype correlation was attempted according to the type and site of the variant found.

## 2. Results

Out of the 502 patients with a clinical diagnosis of neurofibromatosis type I, 108 harbored mutations that had never been previously reported in the international disease databases (Table S1). Among those, 45 were single-base substitutions (26 missense, 10 truncating, 6 splice-site, and 3 synonymous), 44 small deletions, (41 truncating, 2 in-frame, and 1 splice-site), 12 duplications (all producing truncating protein), 5 indels (4 truncating and 1 in-frame), 1 insertion (truncating), and 1 large deletion spanning Exons 2–19 (Table 1).

Truncating variants are the most frequently encountered alterations in our population (69/108; 63.9%), followed by missense (26/108; 24%), splice-site (7/108; 6.5%), synonymous (3/108; 2.8%), and in-frame variants (3/108; 2.8%) (Table 1). Out of the 108 previously undescribed *NF1* alleles, 14 were familial (inherited by one of the two parents presenting signs and symptoms of NF1) and 94 de novo. Twenty-nine (26.9%) variants were of unknown significance (25 missense, 3 synonymous, and 1 in-frame), 16 (14.8%) were likely pathogenic (7 splice-site, 8 truncating, and 1 missense) and 63 (58.3%) variants were pathogenic (61 truncating and 2 in-frame) (Table 2).

**Figure 1:**
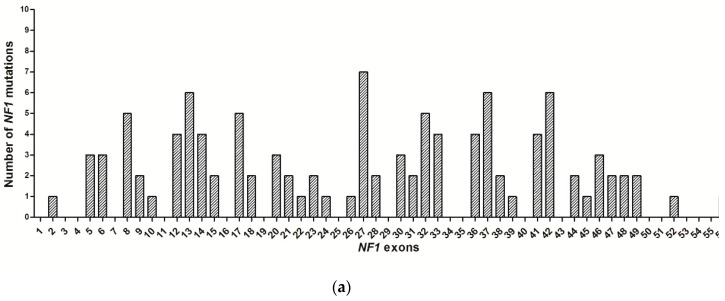

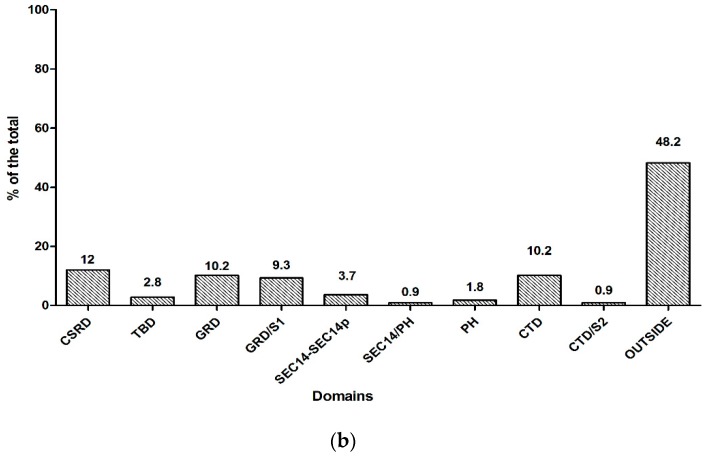
(**a**) Distribution of the *NF1* variants by exons. (**b**) Distribution of the variants in neurofibromin domains. CSRD: cysteine–serine-rich domain; TBD: tubulin-binding domain; GRD: GTPase-activating protein-related domain; S1: syndecan binding domain 1; PH: pleckstrin homology domain; CTD: carboxy-terminal domain; S2: syndecan binding domain 2; SEC14/ SEC14p: Sec14-like lipid binding module.

The distribution of the previously unreported variants in the *NF1* gene showed that no mutational hotspots exist in the gene and that the most frequently hit exons (including the flanking introns, +/−2 bp) were Exons 13, 27, 37 (7 mutations), 8, 32, 42 (6 mutations), and 32 (5 mutations) (Figure 1a). Five out the 108 mutations (4.6%) were located within CpG dinucleotides. When the functional domains of the protein neurofibromin 1 were considered, 13 (12%) mutations were identified in the CSRD domain, 3 (2.8%) in TBD, 11 mutations (10.2%) in GRD, 10 (9.3%) in GRD/S1, 4 (3.7%) in SEC14-SEC14P, 1 (0.9%) in SEC14/PH, 2 (1.8%) in PH, 11 (10.2%) in CTD, and 1 (0.9) in CTD/S2. Furthermore, 52 (48.2%) mutations were localized outside the neurofibromin domains (Figure 1b).

Twenty-nine variants (26.9% of the total) were classified as variants of unknown significance (VUS, Score 3), 16 (14.8%) as likely pathogenetic (Score 4) and 63 (58.3%) as pathogenetic (Score 5). De novo variants were assigned to Classes 4 and 5 in 73.4% (69 out of 94) of cases, whereas the familial ones were attributed to Class 4 or Class 5 in 71.4% (10 out of 14) of cases, χ-square = 0.004, *p* = 1) (Table 2 and Table S2).

**Figure 2:**
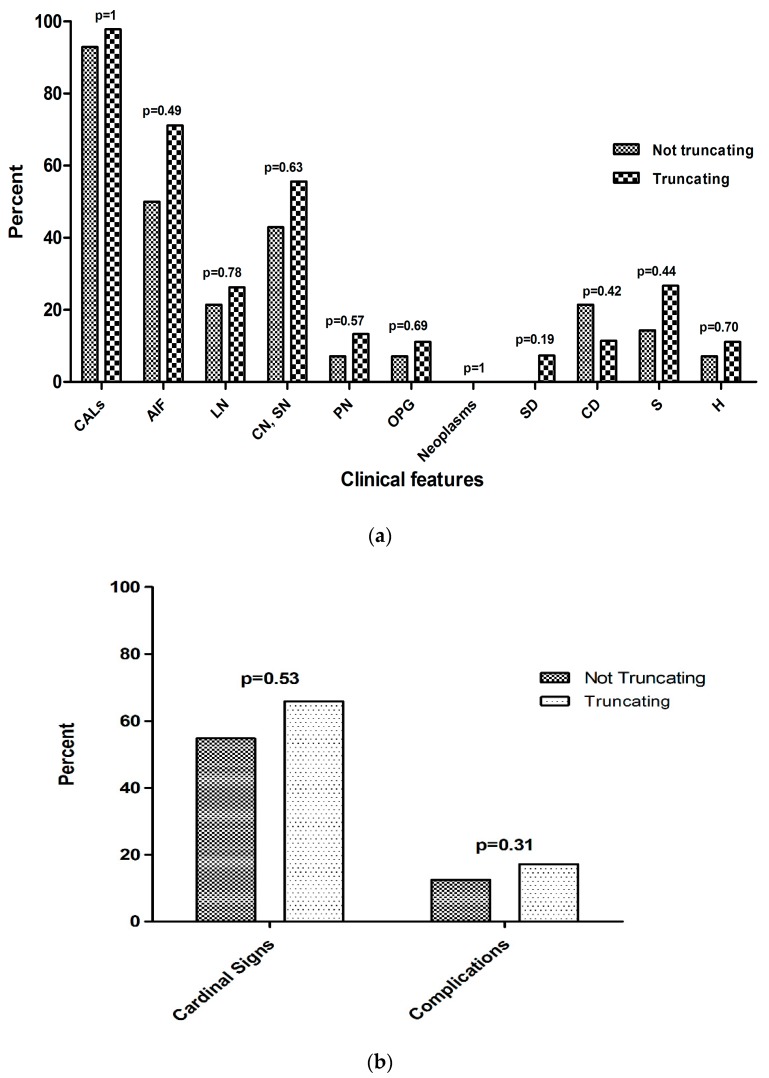
(**a**) Clinical features and (**b)** aggregation of cardinal signs and complications in patients with truncating and non-truncating mutations. CALs: café-au-lait patches; AIF: axillary or groin freckling; LN: Lisch nodules; CN/SN: cutaneous/subcutaneous; PN: plexiform neurofibromas; OPG: optic pathway glioma; SD: sphenoid wing dysplasia; CD: cognitive deficit; S: scoliosis; H: hypertension.

As far as the phenotypic features were concerned (details of each patient are reported in Table S2), a higher proportion of specific NF1 features was found in patients with truncating mutations, compared to patients with non-truncating ones (the 7 splice-site variants were excluded from this analysis due the lack of functional data about their effect on the protein), but none of the differences found reached statistical significance (cafe-au-lait patches (CALs): 97.8% vs. 92.9% for truncating mutations vs. non-truncating mutations, respectively, *p* = 1; axillary or groin freckling (AIF): 71.1% vs. 50%, *p* = 0.49; Lisch nodules (LN): 26.2% vs. 21.4%, *p* = 0.78; cutaneous/subcutaneous neurofibromas (CN/SN): 55.6% vs. 42.9%, *p* = 0.63; plexiform neurofibromas (PN): 13.3% vs. 7.1%, *p* = 0.57; optic pathway glioma (OPG): 11.1% vs. 7.1%, *p* = 0.69; neoplasms: 0% vs. 0%, *p* = 1; sphenoid wing dysplasia (SD): 7.3% vs. 0%, *p* = 0.19; cognitive deficit (CD): 11.4% vs. 21.4%, *p* = 0.42; scoliosis (S): 26.7% vs. 14.3%, *p* = 0.44, hypertension (H): 11.1% vs. 7.1%, *p* = 0.70) (Figure 2a). After the phenotypic features were grouped into cardinal signs (CALs, AIF, and LN) and complications (all the other signs), no significant differences were found between patients with truncating mutations vs. those with non-truncating mutations (17.2% vs. 12.5%, *p* = 0.31) (Figure 2b). The average paternal age in de novo mutations (36 years and 4 months) was higher than that in familial mutations (35 years), but, again, no statistically significant differences were found.

## 3. Discussion

Neurofibromatosis type I is a model genetic disorder due to the high mutation rate of its causative gene, *NF1* [6]. However, despite the long-standing knowledge of the disease, studies of the genotype/phenotype correlation have failed to find clear associations with specific gene variations [13,14], probably due to the tumor suppressor mechanism of the *NF1* gene, which acts in a recessive manner and requires a second random somatic, occult mutational event for its expression [15,16]. For these reasons, the majority of studies have yielded negative results [13,14,17], and very few have reported mutation-specific correlations: a 3 bp in-frame deletion, c.2970_2972delAAT, has been related to the absence of cutaneous neurofibromas [18,19], and missense mutations affecting codon 1809 (Arg1809Cys) have been associated with Noonan-like dysmorphisms and the absence of neurofibromas [20,21]. More general observations have indicated a poorer cognitive prognosis and a higher risk of tumors, neurofibromas, and MPNSTs for the whole *NF1* gene deletions [9,10,11]; other reports have hypothesized an association of splice-site mutations with an increased tendency to develop neoplasms [22], of truncating mutations with the presence of Lisch nodules and CALs [23,24] and of non-truncating mutations with pulmonary stenosis [25]. The results of our study show that, starting from a population of affected patients, there are no hotspots for mutations in the *NF1* gene, nor is there any preferential involvement of specific protein domains. We did not observe any significant correlation between the type of mutation and the phenotypic features, both taken individually (Figure 2a) and grouped together (Figure 2b). Moreover, no malignancies were registered (Figure 2a), possibly due to the young age of the population tested (median age 25 years and 5 months). In conclusion, our data confirm the majority of the previous studies about the weak predictive potential in clinical terms of the mutation found, which cannot be applied in individual cases, especially in predictive counselling settings like the prenatal diagnosis or when formulating the disease prognosis of an affected child.

Another focus of our study was to highlight potential factors involved in the high mutation rate in the *NF1* gene, for which two main risk factors have been advocated: the paternal age of the affected [26] and the genomic context [27], which are the common determinants of the genetic variability at the nucleotide level [28]. Considering our population, the paternal age at birth was slightly higher in de novo mutations (mean age 36 years and 4 months) than in familial ones (mean 35 age), although no statistically significant association was found, also probably due to a type 2 statistical error for the limited number of observations in the familial group (14 cases). The context, i.e., the identity of the nucleotides surrounding the mutation, had clearly no impact on the mutational load, since only 5 of 108 fell into the CpG dinucleotides, the most mutable sequences in humans [27]. Regardless of the cause, the high variability of the gene is evident when querying the ClinVar database [3] for all known *NF1* exonic variants (including splice-site +/− 2 bp): the reported mutations occur, on average, in one out of every eight nucleotides—mostly missense (74%), followed by truncating (20%), and splice-site (6%). They present an inverted ratio compared to our NF1 cohort, in which the most common mutation group is represented by the truncating (63.9%) variations, followed by missense (24%) and splice-site (6.5%) variations. This difference is reflected in the clinical significance of the variant found (for truncating mutations, it is easier to foresee a causal role for the disease), which in the ClinVar database was in 44% of the cases considered as VUS, a ratio that in our cohort was 26.9% (29/108), a lower but still relevant percentage of tests with inconclusive results about pathogenesis.

In conclusion, the present genotype/phenotype correlation study based on 108 previously undescribed *NF1* variants failed to show any gene hotspot for mutation and any significant association of the clinical presentation of the disease with the type of gene mutation found. In at least one fourth of the cases, the clinical significance of the variant found at the genetic testing remains uncertain, with important reflections on the genetic counseling of the patients and their families.

## 4. Materials and Methods

### 4.1. Patients

The analysis is based on the records of Parma University Hospital’s Unit of Medical Genetics, covering the years 2000 to 2017. The laboratory functions as a hub for the entire Emilia Romagna Region (4.5 million inhabitants) and attracts patients from other Italian regions as well. Genetic testing was performed on patients having a clinical suspicion of neurofibromatosis type I based on the presence of at least two of the clinical manifestations proposed by the National Institute of Health Consensus Development Conference, i.e., the presence of six or more CALs >15 mm in adults and >5 mm in children, two or more CN/SN or PN, AIF, LN, OPG, MPNST, PCC, CD, and distinctive osseous lesions such as SD or S [29]. Each sample given to our laboratory was accompanied by a clinical chart in which a clinical description of the patient was reported. Patient’s clinical records, genealogical trees, and genetic test results were all collected and archived in a specifically dedicated Excel file. In the 2000–2017 period, 502 subjects with a clinical suspicion of neurofibromatosis type I were subjected to genetic testing as part of the diagnostic process.

### 4.2. NF1 Genetic Test

From April 2000 to June 2016 (397 out of the total 502 patients, 79.1%), genetic analyses on genomic DNA were conducted using denaturing high pressure liquid chromatography (DHPLC), which exploits the differential retention of homoduplex and heteroduplex DNA under conditions of partial thermal denaturation (reported detection rate for *NF1* mutations: 72% [30]). Primer pairs were designed according to the published reference genomic *NF1* sequence (GenBank accession number NG_009018.1). To characterize the samples with a profile different from a wild-type control, direct sequencing of the fragments was performed using M13 Universal primers and a GenomeLab DTCS Quick Start kit for Dye Cycle Sequencing on a CEQ 2000XL Sequence Analyzer (Beckman Coulter, CA, USA). Starting from June 2016 (105 out of the total 502 patients, 20.9%), the genetic analyses were performed via next-generation sequencing using the Illumina MiSeq (TruSeq Custom Amplicon v.1.5, San Diego, CA, USA) platform (reported detection rate for *NF1* mutations: 88% [15]). For the description and nomenclature of sequence variations at the DNA and protein level, the Mutalyzer software version 2.0.22 [31] was used with the NM_000267.3 reference sequence. Multiplex ligation-dependent probe amplification (MLPA, MRC Holland Amsterdam, The Netherlands) as been used for the detection of deletions or duplications spanning one or more exons in those cases negative at the sequencing analysis. For predicting the pathogenicity of the missense mutations, the REVEL (Rare Exome Variant Ensemble Learner) tool was used, an Ensemble method based on the 13 most commonly used prediction software [32].

### 4.3. The Database

After the consultation of the dedicated international databases (Clinvar [3]; The Human Gene Mutation Database, HGMD [33]; Leiden Open Variation Database 3.0, LOVD [34]), the previously undescribed *NF1* variants together with the genetic information were grouped and prepared in an Excel file. In particular, records have included, for each variant, the type, effect, aminoacid change, exon involved, protein domain, segregation (de novo/familial), and population frequency according to Ensembl [35] and ExAC [36]. All the information has been submitted to the ClinVar [3] gene variant database (Accession number: SUB2798650). Moreover, the main clinical features of the patients with previously undetected mutations were recorded according to the Human Phenotype Ontology (HPO) [37] (Table 3). Finally, the classical 5-tiered system, which classifies the variants on the basis of standard criteria [1] as 1 (benign), 2 (likely benign), 3 (uncertain significance), 4 (likely pathogenic), and 5 (pathogenic) was adopted.

The statistical analysis was performed using the SPSS software (version 10.0, SPSS Corp, Chicago, IL, USA), converting descriptive variables to numerical values and using the chi-square test to evaluate differences in mutation distribution among exons and protein domains of neurofibromin and for genotype–phenotype comparisons between groups. Statistical results were corrected for multiple testing using Holm’s test [38].

The local Institutional Review Board approval was requested and obtained for this study (Prot. N. 0051/2017).

## Figures and Tables

**Table 1:** Classification of the previously undescribed *NF1* mutations.

Type of Mutation, *n*	Missense (%)	Truncating (%)	Splice (%)	Silent (%)	In-Frame (%)
Single Nucleotide Variants, 45	26 (57.8)	10 (22.2)	6 (13.3)	3 (6.7)	0 (0)
Small Deletions, 44	0 (0)	41 (93.2)	1 (2.3)	0 (0)	2 (4.5)
Small Duplications, 12	0 (0)	12 (100)	0 (0)	0 (0)	0 (0)
Indels, 5	0 (0)	4 (80)	0 (0)	0 (0)	1 (20)
Insertions, 1	0 (0)	1 (100)	0 (0)	0 (0)	0 (0)
Large Deletions, 1	0 (0)	1 (100)	0 (0)	0 (0)	0 (0)
Total, 108	26 (24)	69 (63.9)	7 (6.5)	3 (2.8)	3 (2.8)

**Table 2:** Classification of the *NF1* mutations according with pathogenicity score.

Mutation Type, *n* (%)	Pathogenity Score, *n* (%)				
	1	2	3	4	5
Missense, 26 (24)	0 (0)	0 (0)	25 (96.1)	1 (3.9)	0 (0)
Truncating, 69 (63.9)	0 (0)	0 (0)	0 (0)	8 (11.6)	61 (88.4)
Splicing, 7 (6.5)	0 (0)	0 (0)	0 (0)	7 (100)	0 (0)
Silent, 3 (2.8)	0 (0)	0 (0)	3 (100)	0 (0)	0 (0)
In-frame, 3 (2.8)	0 (0)	0 (0)	1 (33.3)	0 (0)	2 (66.7)
Total, 108	0 (0)	0 (0)	29 (26.9)	16 (14.8)	63 (58.3)

**Table 3:** Clinical features of patients with novel *NF1* mutation.

Clinical Features (HPO Code)	Number of Patients with Known Status	Number of Patients with Positive Status (%)
Café-au-lait spots (0000957)	59	57/59 (96.6)
Axillary or inguinal freckles (0000997, 0030052)	59	39/59 (66.1)
Lisch nodules (0009737)	56	14/56 (25)
Cutaneous, subcutaneous neurofibromas (0100698, 0100698)	59	31/59 (52.5)
Plexiform neurofibromas (0009732)	59	7/59 (11.9)
Optic pathway glioma (0009734)	59	6/59 (10.2)
Neoplasms (0002664)	59	0/59 (0)
Sphenoid-shank dysplasia (0000924)	55	3/55 (5.4)
Cognitive defects (0100543)	58	8/58 (13.8)
Scoliosis (0002650)	59	14/59 (23.7)
Hypertension (0000822)	59	6/59 (10.2)

## Supplementary Materials

Supplementary materials can be found at www.mdpi.com/1422-0067/18/10/2071/s1.
