# Supplementary material for: Patterns of Novel Alleles and Genotype/Phenotype Correlations Resulting from the Analysis of 108 Previously Undetected Mutations in Patients Affected by Neurofibromatosis Type I
Source: Int J Mol Sci. 2017 Sep 29;18(10):2071. doi: 10.3390/ijms18102071 (PMC5666753; doi:10.3390/ijms18102071)
Supplement: Supplementary file 1 [file ijms-18-02071-s001.pdf]

Table S1. List of the previously undescribed *NF1* variants under study (*N* = 108).

ID	Exon	Nucleotide Change	Affected Protein	Variant Type	Predicted Effect	Allele Frequency		REVEL Score (0 to 1)	Pathogenicity Score	Familial (F)/De Novo (N)	Gender (M/F)	Domain
						ExAC	IGSR					
1	2	Deletion exons 2–19	p.?	deletion	truncation	NP	NP		5	N	M	Outside
2	5	c.538_541del	p.Leu180Serfs*10	deletion	truncation	NP	NP		5	F	M	Outside
3	5	c.541_542del	p.Gln181Valfs*19	deletion	truncation	NP	NP		4	N	M	Outside
4	5	c.586+2T>C	p.?	substitution	splicing	NP	NP		4	F	F	Outside
5	6	c.603del	p.Phe201Leufs*4	deletion	truncation	NP	NP		5	N	F	Outside
6	6	c.610dup	p.Leu204Profs*12	duplication	truncation	NP	NP		5	N	M	Outside
7	6	c.615G>A	p.=	substitution	synonymous	NP	NP		3	N	F	Outside
8	8	c.789del	p.Ala264Glnfs*17	deletion	truncation	NP	NP		5	N	M	Outside
9	8	c.792del	p.Ala265Glnfs*16	deletion	truncation	NP	NP		5	N	M	Outside
10	8	c.862del	p.Val288Trpfs*7	deletion	truncation	NP	NP		4	N	M	Outside
11	8	c.886A>T	p.Lys296*	substitution	truncation	NP	NP		4	N	M	Outside
12	8	c.888+1G>C	p.?	substitution	splicing	NP	NP		4	F	F	Outside
13	9	c.1041_1045del	p.Gln347Hisfs*4	deletion	truncation	NP	NP		5	N	F	Outside
14	9	c.1061A>G	p.Lys354Arg	substitution	missense	NP	NP	0.309	3	N	M	Outside
15	10	c.1144del	p.Ser382Leufs*5	deletion	truncation	NP	NP		4	N	M	Outside
16	12	c.1280_1292del	p.Pro427Leufs*42	deletion	truncation	NP	NP		5	N	F	Outside
17	12	c.1324_1325del	p.Met442Valfs*3	deletion	truncation	NP	NP		5	N	M	Outside
18	12	c.1374dup	p.Ala459Serfs*11	duplication	truncation	NP	NP		5	N	M	Outside
19	12	c.1392del	p.Ser465Valfs*8	deletion	truncation	NP	NP		5	F	F	Outside
20	13	c.1400del	p.Thr467Asnfs*6	deletion	truncation	NP	NP		5	N	M	Outside
21	13	c.1460G>A	p.Arg487Lys	substitution	missense	NP	NP	0.107	3	N	M	Outside
22	13	c.1462del	p.Ser488Alafs*10	deletion	truncation	NP	NP		5	N	M	Outside
23	13	c.1469_1472del	p.Lys490Ilefs*7	deletion	truncation	NP	NP		5	N	F	Outside
24	13	c.1477C>G	p.Leu493Val	substitution	missense	NP	NP	0.214	3	N	M	Outside
25	13	c.1525dup	p.Cys509Leufs*2	duplication	truncation	NP	NP		5	N	F	Outside
26	14	c.1561del	p.Ser521Valfs*5	deletion	truncation	NP	NP		5	N	M	Outside
27	14	c.1585C>T	p.Leu529Phe	substitution	missense	NP	NP	0.434	3	N	F	Outside
28	14	c.1613delT	Met538Serfs*18	deletion	truncation	NP	NP		5	F	M	Outside
29	14	c.1641+2delT	p.?	deletion	splicing	NP	NP		4	N	F	CSRD
30	15	c.1683G>A	p.Trp561*	substitution	truncation	NP	NP		5	N	F	CSRD
31	15	c.1714_1721+5del	p.?	deletion	truncation	NP	NP		4	N	M	CSRD
32	17	c.1866T>A	p.Cys622*	substitution	truncation	NP	NP		5	N	M	CSRD
33	17	c.1883_1885delinsCC	p.Tyr628Serfs*3	indel	truncation	NP	NP		5	N	F	CSRD
34	17	c.1889T>A	p.Val630Glu	substitution	missense	NP	NP	0.202	3	F	M	CSRD
35	17	c.1918dup	p.Thr640Asnfs*9	duplication	truncation	NP	NP		5	N	M	CSRD
36	17	c.1949T>A	p.Leu650*	substitution	truncation	NP	NP		5	N	F	CSRD
37	18	c.2034delinsCA	p.Ile679Asnfs*21	indel	truncation	NP	NP		5	N	F	CSRD
38	18	c.2218G>T	p.Glu740*	substitution	truncation	NP	NP		5	N	F	CSRD
39	20	c.2338_2343del	p.Thr780_His781del	deletion	in frame	NP	NP		5	N	M	CSRD

ID	Exon	Nucleotide Change	Affected Protein	Variant Type	Predicted Effect	Allele Frequency		REVEL Score (0 to 1)	Pathogenicity Score	Familial (F)/De Novo (N)	Gender (M/F)	Domain
						ExAC	IGSR					
40	20	c.2349del	p.Lys783Asnfs*8	deletion	truncation	NP	NP		5	N	M	CSRD
41	20	C.2392A>C	p.Lys798Gln	substitution	missense	NP	NP	0.298	4	F	M	CSRD
42	21	c.2764G>A	p.Gly922Ser	substitution	missense	NP	NP	0.861	3	N	M	Outside
43	21	c.2848del	p.Gln950Argfs*4	deletion	truncation	NP	NP		5	N	F	Outside
44	22	c.2886_2897 del	p.Glu962_Ala966delinsAsp	indel	in frame	NP	NP		3	N	F	Outside
45	23	c.3027del	p.Gln1010Lysfs*2	deletion	truncation	NP	NP		5	N	F	Outside
46	23	c.3040A>T	p.Lys1014*	substitution	truncation	NP	NP		5	N	M	Outside
47	24	c.3189T>A	p.Cys1063*	substitution	truncation	NP	NP		5	N	M	Outside
48	26	c.3429_3432dup	p.Thr1145Leufs*51	duplication	truncation	NP	NP		5	N	F	TBD
49	27	c.3521A>G	p.Gln1174Arg	substitution	missense	NP	NP	0.431	3	N	M	TBD
50	27	c.3578T>C	p.Phe1193Ser	substitution	missense	NP	NP	0.691	3	N	M	TBD
51	27	c.3591dup	p.Glu1198Argfs*6	duplication	truncation	NP	NP		5	N	F	GRD-GTPase
52	27	c.3632T>G	p.Leu1211Arg	substitution	missense	NP	NP	0.914	3	N	F	GRD-GTPase
53	27	c.3651T>G	p.Asp1217Glu	substitution	missense	NP	NP	0.606	3	F	F	GRD-GTPase
54	27	c.3665del	p.Pro1222Leufs*2	deletion	truncation	NP	NP		5	N	M	GRD-GTPase
55	27	c.3692_3708del	p.Val1231Glyfs*2	deletion	truncation	NP	NP		5	N	M	GRD-GTPase
56	28	c.3732dup	p.Thr1245Tyrfs*4	duplication	truncation	NP	NP		5	N	M	GRD-GTPase
57	28	c.3834C>G	p.Asn1278Lys	substitution	missense	NP	NP	0.579	3	N	M	GRD-GTPase
58	30	c.4000del	p.Glu1334Lysfs*9	deletion	truncation	NP	NP		5	F	M	GRD-GTPase
59	30	c.4109A>C	p.Gln1370Pro	substitution	missense	NP	NP	0.925	3	N	F	GRD-GTPase-S1
60	30	c.4110G>C	p.Gln1370His	substitution	missense	NP	NP	0.771	3	N	F	GRD-GTPase-S1
61	31	c.4261del	p.Met1421Cysfs*27	deletion	truncation	NP	NP		5	N	F	GRD-GTPase-S1
62	31	c.4269+1G>C	p.?	substitution	splicing	NP	NP		4	N	M	GRD-GTPase-S1
63	32	c.4276C>T	p.Gln1426*	substitution	truncation	NP	NP		5	N	M	GRD-GTPase-S1
64	32	c.4309G>A	p.Glu1437Lys	substitution	missense	NP	NP	0.845	3	N	M	GRD-GTPase-S1
65	32	c.4318_4319dup	p.Met1440Ilefs*9	duplication	truncation	NP	NP		5	N	M	GRD-GTPase-S1
66	32	c.4319T>C	p.Met1440Thr	substitution	missense	1.24×10^{-5}	NP	0.952	3	F	M	GRD-GTPase-S1
67	32	c.4340T>G	p.Val1447Gly	substitution	missense	NP	NP	0.863	3	N	F	GRD-GTPase-S1
68	33	c.4397del	p.Pro1466Leufs*6	deletion	truncation	NP	NP		5	N	F	GRD-GTPase-S1
69	33	c.4435A>G	p.Ser1479Gly	substitution	missense	NP	NP	0.467	3	N	M	GRD-GTPase
70	33	c.4457T>G	p.Leu1486*	substitution	truncation	NP	NP		5	N	M	GRD-GTPase
71	33	c.4469T>G	p.Leu1490Arg	substitution	missense	NP	NP	0.934	3	N	F	GRD-GTPase

ID	Exon	Nucleotide Change	Affected Protein	Variant Type	Predicted Effect	Allele Frequency		REVEL Score (0 to 1)	Pathogenicity Score	Familial (F)/De Novo (N)	Gender (M/F)	Domain
						ExAC	IGSR					
72	36	c.4866G>T	p.=	substitution	synonymous	NP	NP		3	N	F	SEC14-SEC14p
73	36	c.4870del	Thr1625Profs*52	deletion	truncation	NP	NP		5	N	F	SEC14-SEC14p
74	36	c.4935dup	p.Pro1646Serfs*15	insertion	truncation	NP	NP		5	N	M	SEC14-SEC14p
75	36	c.4973_4978del	p.Ile1658_Tyr1659del	deletion	in frame	NP	NP		5	N	F	SEC14-SEC14p
76	37	c.5206-1G>C	p.?	substitution	splicing	NP	NP		4	N	M	SEC14-SEC14p-PH
77	37	c.5322T>A	p.Asp1774Glu	substitution	missense	NP	NP	0.651	3	N	M	PH
78	37	c.5413C>G	p.His1805Asp	substitution	missense	NP	NP	0.761	3	N	M	PH
79	37	c.5483_5490del	p.Asp1828Glyfs*10	deletion	truncation	NP	NP		5	N	M	Outside
80	37	c.5508delC	p.Ile1836Metfs*6	deletion	truncation	NP	NP		4	N	F	Outside
81	37	c.5508_5509delinsT	p.Ala1837Hisfs*5	indel	truncation	NP	NP		5	N	M	Outside
82	38	c.5574del	p.Leu1859*	deletion	truncation	NP	NP		5	N	M	Outside
83	38	c.5609dup	p.Leu1871Valfs*21	duplication	truncation	NP	NP		5	N	F	Outside
84	39	c.5780dup	p.Tyr1927*	duplication	truncation	NP	NP		5	F	F	Outside
85	41	c.6134delC	p.Thr2045Ilefs*4	deletion	truncation	NP	NP		5	N	F	Outside
86	41	c.6148C>T	p.Gln2050*	substitution	truncation	NP	NP		5	F	M	Outside
87	41	c.6263del	p.Phe2088Serfs*2	deletion	truncation	NP	NP		5	N	F	Outside
88	41	c.6361A>C	p.Ser2121Arg	substitution	missense	NP	NP	0.796	3	N	M	Outside
89	42	c.6365-2A>C	p.?	substitution	splicing	NP	NP		4	N	F	Outside
90	42	c.6389_6393delinsA	p.Leu2130Hisfs*2	indel	truncation	NP	NP		5	N	F	Outside
91	42	c.6399dup	p.Glu2134Argfs*14	duplication	truncation	NP	NP		5	N	F	Outside
92	42	c.6482del	p.Tyr2161Serfs*18	deletion	truncation	NP	NP		5	N	F	Outside
93	42	c.6483_6487del	p.Tyr2161*	deletion	truncation	NP	NP		5	F	F	Outside
94	42	c.6537del	p.Ser2180Profs*17	deletion	truncation	NP	NP		4	N	M	Outside
95	44	c.6747del	p.Ser2251Alafs*8	deletion	truncation	NP	NP		4	N	F	Outside
96	44	c.6756G>T	p.Lys2252Asn	substitution	missense	NP	NP	0.229	3	N	F	Outside
97	45	c.6815del	p.Ala2272Valfs*3	deletion	truncation	NP	NP		5	N	F	CTD
98	46	c.6915T>C	p.=	substitution	synonymous	NP	NP		3	N	F	CTD
99	46	c.6967del	p.Thr2323Leufs*2	deletion	truncation	NP	NP		5	N	F	CTD
100	46	c.6999+2T>C	p.?	substitution	splicing	NP	NP		4	N	F	CTD
101	47	c.7118T>G	p.Leu2373Arg	substitution	missense	NP	NP	0.708	3	F	M	CTD
102	47	c.7126G>A	p.Gly2376Arg	substitution	missense	NP	NP	0.739	3	N	F	CTD
103	48	c.7197dup	p.Asn2400*	duplication	truncation	NP	NP		5	N	M	CTD
104	48	c.7224del	p.Phe2408Leufs*3	deletion	truncation	NP	NP		5	N	F	CTD
105	49	c.7274_7275del	p.Ser2425*	deletion	truncation	NP	NP		5	N	F	CTD
106	49	c.7320del	p.Leu2441Phefs*27	deletion	truncation	NP	NP		5	N	F	CTD
107	52	c.7719del	p.Val2575Phefs*28	deletion	truncation	NP	NP		5	N	M	CTD
108	56	c.8113G>A	p.Asp2705Asn	substitution	missense	NP	NP	0.148	3	N	M	CTD-S2

Legend: CSRD:cysteine–serine-rich domain; TBD: tubulin-binding domain; GRD: GTPase-activating protein-related domain; SH1: syndecan binding domain 1; PH: pleckstrin homology domain; CTD: carboxy-terminal domain; SH2: syndecan binding domain 2; ExAC: The Exome Aggregation Consortium; IGSR: International Genome Sample Resource and the 1000 genomes browser; NP: not provided. REVEL (Rare Exome Variant Ensemble Learner) score, the ensamble prediction tool used for the study, refers only to missense variants, with a deleterious score cutoff >0.5

Table S2. Clinical phenotype of the *NF1* mutations under study.

ID	Exon	Nucleotide Change	Affected Protein	Pathogenicity Score	Gender (M/F)	CALs	AIF	LN	CN SN	PN	OPG	Neoplasms	SD	CD	S	H
1	2	Deletion exons 2–19	p.?	5	M	Y	Y	N	N	N	Y	N	NA	NA	N	N
2	5	c.538_541del	p.Leu180Serfs*10	5	M	NA	NA	NA	NA	NA	NA	NA	NA	NA	NA	NA
3	5	c.541_542del	p.Gln181Valfs*19	4	M	NA	NA	NA	NA	NA	NA	NA	NA	NA	NA	NA
4	5	c.586+2T>C	p.?	4	F	NA	NA	NA	NA	NA	NA	NA	NA	NA	NA	NA
5	6	c.603del	p.Phe201Leufs*4	5	F	Y	Y	N	Y	Y	N	N	NA	N	N	N
6	6	c.610dup	p.Leu204Profs*12	5	M	Y	Y	N	Y	N	N	N	N	N	Y	N
7	6	c.615G>A	p.=	3	F	NA	NA	NA	NA	NA	NA	NA	NA	NA	NA	NA
8	8	c.789del	p.Ala264Glnfs*17	5	M	NA	NA	NA	NA	NA	NA	NA	NA	NA	NA	NA
9	8	c.792del	p.Ala265Glnfs*16	5	M	NA	NA	NA	NA	NA	NA	NA	NA	NA	NA	NA
10	8	c.862del	p.Val288Trpfs*7	4	M	Y	Y	N	N	N	N	N	N	N	N	N
11	8	c.886A>T	p.Lys296*	4	M	Y	Y	N	N	N	N	N	N	N	N	N
12	8	c.888+1G>C	p.?	4	F	Y	Y	NA	Y	N	N	N	Y	N	N	N
13	9	c.1041_1045del	p.Gln347Hisfs*4	5	F	Y	N	Y	Y	N	N	N	N	N	Y	N
14	9	c.1061A>G	p.Lys354Arg	3	M	NA	NA	NA	NA	NA	NA	NA	NA	NA	NA	NA
15	10	c.1144del	p.Ser382Leufs*5	4	M	Y	Y	N	N	N	Y	N	N	N	N	N
16	12	c.1280_1292del	p.Pro427Leufs*42	5	F	NA	NA	NA	NA	NA	NA	NA	NA	NA	NA	NA
17	12	c.1324_1325del	p.Met442Valfs*3	5	M	Y	Y	Y	N	N	N	N	Y	N	N	N
18	12	c.1374dup	p.Ala459Serfs*11	5	M	Y	Y	N	N	N	N	N	Y	N	N	Y
19	12	c.1392del	p.Ser465Valfs*8	5	F	Y	Y	Y	Y	N	N	N	N	Y	Y	N
20	13	c.1400del	p.Thr467Asnfs*6	5	M	NA	NA	NA	NA	NA	NA	NA	NA	NA	NA	NA
21	13	c.1460G>A	p.Arg487Lys	3	M	NA	NA	NA	NA	NA	NA	NA	NA	NA	NA	NA
22	13	c.1462del	p.Ser488Alafs*10	5	M	Y	N	N	Y	N	Y	N	N	Y	N	Y
23	13	c.1469_1472del	p.Lys490Ilefs*7	5	F	Y	Y	Y	N	N	N	N	N	Y	Y	N
24	13	c.1477C>G	p.Leu493Val	3	M	NA	NA	NA	NA	NA	NA	NA	NA	NA	NA	NA
25	13	c.1525dup	p.Cys509Leufs*2	5	F	Y	Y	N	N	N	N	N	N	N	N	N
26	14	c.1561del	p.Ser521Valfs*5	5	M	Y	Y	NA	Y	Y	N	N	N	N	N	N
27	14	c.1585C>T	p.Leu529Phe	3	F	Y	Y	N	Y	N	N	N	N	Y	Y	N
28	14	c.1613delT	Met538Serfs*18	5	M	NA	NA	NA	NA	NA	NA	NA	NA	NA	NA	NA
29	14	c.1641+2delT	p.?	4	F	Y	N	N	N	N	N	N	NA	N	N	N
30	15	c.1683G>A	p.Trp561*	5	F	NA	NA	NA	NA	NA	NA	NA	NA	NA	NA	NA
31	15	c.1714_1721+5del	p.?	4	M	NA	NA	NA	NA	NA	NA	NA	NA	NA	NA	NA
32	17	c.1866T>A	p.Cys622*	5	M	NA	NA	NA	NA	NA	NA	NA	NA	NA	NA	NA
33	17	c.1883_1885delinsCC	p.Tyr628Serfs*3	5	F	Y	Y	N	Y	N	N	N	N	N	N	N
34	17	c.1889T>A	p.Val630Glu	3	M	Y	Y	N	N	N	Y	N	N	N	N	N
35	17	c.1918dup	p.Thr640Asnfs*9	5	M	Y	Y	N	N	N	N	N	NA	Y	N	N

ID	Exon	Nucleotide Change	Affected Protein	Pathogenicity Score	Gender (M/F)	CALs	AIF	LN	CN SN	PN	OPG	Neoplasms	SD	CD	S	H
36	17	c.1949T>A	p.Leu650*	5	F	NA	NA	NA	NA	NA	NA	NA	NA	NA	NA	NA
37	18	c.2034delinsCA	p.Ile679Asnfs*21	5	F	Y	Y	N	N	N	N	N	N	N	N	N
38	18	c.2218G>T	p.Glu740*	5	F	Y	Y	N	N	N	N	N	N	N	N	N
39	20	c.2338_2343del	p.Thr780_His781del	5	M	NA	NA	NA	NA	NA	NA	NA	NA	NA	NA	NA
40	20	c.2349del	p.Lys783Asnfs*8	5	M	NA	NA	NA	NA	NA	NA	NA	NA	NA	NA	NA
41	20	C.2392A>C	p.Lys798Gln	4	M	Y	N	N	N	N	N	N	N	N	N	N
42	21	c.2764G>A	p.Gly922Ser	3	M	Y	Y	Y	N	N	N	N	N	N	N	N
43	21	c.2848del	p.Gln950Argfs*4	5	F	Y	N	N	Y	Y	N	N	N	N	Y	N
44	22	c.2886_2897 del	p.Glu962_Ala966delinsAsp	3	F	NA	NA	NA	NA	NA	NA	NA	NA	NA	NA	NA
45	23	c.3027del	p.Gln1010Lysfs*2	5	F	Y	Y	N	N	N	N	N	NA	N	N	N
46	23	c.3040A>T	p.Lys1014*	5	M	NA	NA	NA	NA	NA	NA	NA	NA	NA	NA	NA
47	24	c.3189T>A	p.Cys1063*	5	M	Y	Y	N	Y	N	N	N	N	N	Y	N
48	26	c.3429_3432dup	p.Thr1145Leufs*51	5	F	NA	NA	NA	NA	NA	NA	NA	NA	NA	NA	NA
49	27	c.3521A>G	p.Gln1174Arg	3	M	NA	NA	NA	NA	NA	NA	NA	NA	NA	NA	NA
50	27	c.3578T>C	p.Phe1193Ser	3	M	Y	N	N	N	N	N	N	N	N	N	N
51	27	c.3591dup	p.Glu1198Argfs*6	5	F	Y	N	N	N	N	N	N	N	N	N	N
52	27	c.3632T>G	p.Leu1211Arg	3	F	NA	NA	NA	NA	NA	NA	NA	NA	NA	NA	NA
53	27	c.3651T>G	p.Asp1217Glu	3	F	Y	N	N	N	N	N	N	N	N	N	N
54	27	c.3665del	p.Pro1222Leufs*2	5	M	Y	Y	N	N	N	N	N	N	N	Y	N
55	27	c.3692_3708del	p.Val1231Glyfs*2	5	M	Y	Y	Y	Y	N	N	N	N	N	N	N
56	28	c.3732dup	p.Thr1245Tyrfs*4	5	M	Y	Y	N	Y	N	N	N	N	N	Y	N
57	28	c.3834C>G	p.Asn1278Lys	3	M	NA	NA	NA	NA	NA	NA	NA	NA	NA	NA	NA
58	30	c.4000del	p.Glu1334Lysfs*9	5	M	Y	N	N	N	N	N	N	N	N	N	N
59	30	c.4109A>C	p.Gln1370Pro	3	F	NA	NA	NA	NA	NA	NA	NA	NA	NA	NA	NA
60	30	c.4110G>C	p.Gln1370His	3	F	NA	NA	NA	NA	NA	NA	NA	NA	NA	NA	NA
61	31	c.4261del	p.Met1421Cysfs*27	5	F	Y	N	N	Y	N	N	N	N	N	N	Y
62	31	c.4269+1G>C	p.?	4	M	Y	N	Y	Y	N	Y	N	N	N	N	N
63	32	c.4276C>T	p.Gln1426*	5	M	Y	N	NA	Y	N	N	N	N	Y	N	N
64	32	c.4309G>A	p.Glu1437Lys	3	M	NA	NA	NA	NA	NA	NA	NA	NA	NA	NA	NA
65	32	c.4318_4319dup	p.Met1440Ilefs*9	5	M	NA	NA	NA	NA	NA	NA	NA	NA	NA	NA	NA
66	32	c.4319T>C	p.Met1440Thr	3	M	Y	N	N	Y	N	N	N	N	N	N	N
67	32	c.4340T>G	p.Val1447Gly	3	F	NA	NA	NA	NA	NA	NA	NA	NA	NA	NA	NA
68	33	c.4397del	p.Pro1466Leufs*6	5	F	Y	Y	N	Y	Y	N	N	N	N	N	N
69	33	c.4435A>G	p.Ser1479Gly	3	M	Y	Y	N	Y	Y	N	N	N	N	N	Y
70	33	c.4457T>G	p.Leu1486*	5	M	Y	Y	Y	Y	N	N	N	N	N	N	N
71	33	c.4469T>G	p.Leu1490Arg	3	F	NA	NA	NA	NA	NA	NA	NA	NA	NA	NA	NA
72	36	c.4866G>T	p.=	3	F	Y	Y	N	N	N	N	N	N	N	N	N
73	36	c.4870del	Thr1625Profs*52	5	F	Y	Y	Y	Y	N	N	N	N	N	Y	N
74	36	c.4935dup	p.Pro1646Serfs*15	5	M	Y	Y	N	Y	N	N	N	N	N	N	N
75	36	c.4973_4978del	p.Ile1658_Tyr1659del	5	F	NA	NA	NA	NA	NA	NA	NA	NA	NA	NA	NA
76	37	c.5206-1G>C	p.?	4	M	NA	NA	NA	NA	NA	NA	NA	NA	NA	NA	NA
77	37	c.5322T>A	p.Asp1774Glu	3	M	N	Y	N	Y	N	N	N	N	Y	N	N
78	37	c.5413C>G	p.His1805Asp	3	M	NA	NA	NA	NA	NA	NA	NA	NA	NA	NA	NA

ID	Exon	Nucleotide Change	Affected Protein	Pathogenicity Score	Gender (M/F)	CALs	AIF	LN	CN SN	PN	OPG	Neoplasms	SD	CD	S	H
79	37	c.5483_5490del	p.Asp1828Glyfs*10	5	M	Y	Y	N	Y	N	N	N	N	N	N	N
80	37	c.5508delC	p.Ile1836Metfs*6	4	F	Y	Y	Y	Y	N	N	N	N	N	N	N
81	37	c.5508_5509delinsT	p.Ala1837Hisfs*5	5	M	NA	NA	NA	NA	NA	NA	NA	NA	NA	NA	NA
82	38	c.5574del	p.Leu1859*	5	M	NA	NA	NA	NA	NA	NA	NA	NA	NA	NA	NA
83	38	c.5609dup	p.Leu1871Valfs*21	5	F	NA	NA	NA	NA	NA	NA	NA	NA	NA	NA	NA
84	39	c.5780dup	p.Tyr1927*	5	F	Y	Y	N	Y	N	N	N	N	N	N	Y
85	41	c.6134delC	p.Thr2045Ilefs*4	5	F	Y	Y	N	N	N	Y	N	N	N	N	N
86	41	c.6148C>T	p.Gln2050*	5	M	NA	NA	NA	NA	NA	NA	NA	NA	NA	NA	NA
87	41	c.6263del	p.Phe2088Serfs*2	5	F	NA	NA	NA	NA	NA	NA	NA	NA	NA	NA	NA
88	41	c.6361A>C	p.Ser2121Arg	3	M	Y	N	Y	N	N	N	N	N	Y	Y	N
89	42	c.6365-2A>C	p.?	4	F	Y	Y	N	Y	N	N	N	N	N	Y	N
90	42	c.6389_6393delinsA	p.Leu2130Hisfs*2	5	F	Y	N	Y	Y	Y	Y	N	N	N	Y	Y
91	42	c.6399dup	p.Glu2134Argfs*14	5	F	NA	NA	NA	NA	NA	NA	NA	NA	NA	NA	NA
92	42	c.6482del	p.Tyr2161Serfs*18	5	F	N	N	Y	Y	N	N	N	N	N	N	N
93	42	c.6483_6487del	p.Tyr2161*	5	F	NA	NA	NA	NA	NA	NA	NA	NA	NA	NA	NA
94	42	c.6537del	p.Ser2180Profs*17	4	M	Y	Y	Y	Y	N	N	N	N	N	N	N
95	44	c.6747del	p.Ser2251Alafs*8	4	F	Y	N	NA	N	N	N	N	N	N	N	N
96	44	c.6756G>T	p.Lys2252Asn	3	F	NA	NA	NA	NA	NA	NA	NA	NA	NA	NA	NA
97	45	c.6815del	p.Ala2272Valfs*3	5	F	Y	N	N	N	N	N	N	N	N	N	N
98	46	c.6915T>C	p.=	3	F	NA	NA	NA	NA	NA	NA	NA	NA	NA	NA	NA
99	46	c.6967del	p.Thr2323Leufs*2	5	F	Y	N	N	Y	N	N	N	N	N	Y	N
100	46	c.6999+2T>C	p.?	4	F	Y	N	Y	Y	N	N	N	N	N	N	N
101	47	c.7118T>G	p.Leu2373Arg	3	M	Y	Y	N	Y	N	N	N	N	N	N	N
102	47	c.7126G>A	p.Gly2376Arg	3	F	Y	N	Y	Y	N	N	N	N	N	N	N
103	48	c.7197dup	p.Asn2400*	5	M	NA	NA	NA	NA	NA	NA	NA	NA	NA	NA	NA
104	48	c.7224del	p.Phe2408Leufs*3	5	F	Y	Y	N	Y	Y	N	N	Y	N	Y	N
105	49	c.7274_7275del	p.Ser2425*	5	F	Y	N	N	N	N	N	N	N	N	N	N
106	49	c.7320del	p.Leu2441Phefs*27	5	F	NA	NA	NA	NA	NA	NA	NA	NA	NA	NA	NA
107	52	c.7719del	p.Val2575Phefs*28	5	M	Y	Y	N	N	N	N	N	N	N	N	N
108	56	c.8113G>A	p.Asp2705Asn	3	M	Y	N	N	N	N	N	N	N	N	N	N

Legend: CALs: cafe au lait patches; AIF: axillary or groin freckling; LN: Lisch nodules; CN/SN: cutaneous/subcutaneous; PN: plexiform neurofibromas; OPG: optic pathway glioma; SD: sphenoid wing dysplasia; S: scoliosis; H: hypertension; CD: cognitive deficit; Y: yes; N: no; NA: not available.
